# Dysfunction in primate dorsolateral prefrontal area 46 affects motivation and anxiety

**DOI:** 10.1126/science.adx4142

**Published:** 2025-08-21

**Authors:** Christian M Wood, Rana Banai Tizkar, Martina Fort, Xinhu Zhang, Kevin G Mulvihill, Naixuan Liao, Gemma J Cockcroft, Lauren B McIver, Stephen J Sawiak, Angela C Roberts

**Affiliations:** 1 Department of Physiology, Development and Neuroscience, University of Cambridge; Cambridge, UK; 2 Girton College, University of Cambridge; Cambridge, UK

## Abstract

Dorsolateral prefrontal cortex (dlPFC) is a higher-order brain structure targeted for non-invasive stimulation for treatment resistant depression. Nonetheless, its causal role in emotion regulation is unknown. Here, we discovered that inactivating dlPFC area 46 in marmosets blunts appetitive motivation and heightens threat reactivity. The effects were asymmetric, dependent on the left hemisphere only and were mediated through projections to pregenual cingulate area 32. The antidepressant ketamine blocked the appetitive motivational deficits through mechanisms within subcallosal cingulate area 25 – an area linked with treatment success in dlPFC non-invasive stimulation. Our data uncover an integrated prefrontal network for area 46 that contributes to positive and negative emotion regulation that may be core to our understanding of symptoms and therapeutic strategies for treatment resistant depression and anxiety.

The dorsolateral prefrontal cortex (dlPFC) is considered part of a network involved in higher-order processes, such as attention, abstract thoughts and consciousness (1, 2). This region is known to be involved in the mental representation of information (3), such as in working memory, and provides a capacity for regulating different types of thoughts through inhibitory control mechanisms (4). It is also a target for non-invasive brain stimulation for the treatment of refractory depression (5, 6). DlPFC transcranial magnetic stimulation (TMS) can improve depressive and comorbid anxiety symptoms (5, 7), alongside normalising hyperactivity in another brain region, the subcallosal cingulate cortex (scACC) including area 25 (A25), known to be both overactive in depression and itself a target for deep brain stimulation for treating refractory depression (8–11). Indeed, better treatment responses are observed from dlPFC stimulation sites, such as area 46 (A46), that are more anti-correlated with scACC activity (12, 13), whilst patients responding to scACC deep brain stimulation show increased activity in the dlPFC alongside reductions in scACC activity (8). In addition, functional asymmetry has been observed across the left and right dlPFC, with a recent framework positing hemispheric differences in affective and non-affective processing of information (7), with antidepressant responses primarily associated with the left hemisphere and inhibitory control processes with the right hemisphere (4).

The antidepressant effects of activating the dlPFC are consistent with reductions in activity within dlPFC reported in subjects with depression, as revealed by both resting state and task based functional MRI (14, 15; rs-fMRI and fMRI, respectively). However, what are the neurobiological mechanisms by which dlPFC can regulate positive and negative emotion? Certainly, correlative human neuroimaging studies have implicated dlPFC in the generation and regulation of emotion via processes such as cognitive reappraisal (16), distraction (17) and suppression of both negative stimuli (18) and emotional memories (19). However, its causal involvement in emotion regulation and its functional interaction with A25 of the scACC and other prefrontal and cingulate structures remains unknown.

## Appetitive motivation is reduced by A46 inactivation which can be ameliorated by ketamine in A25

**Fig. 1:**
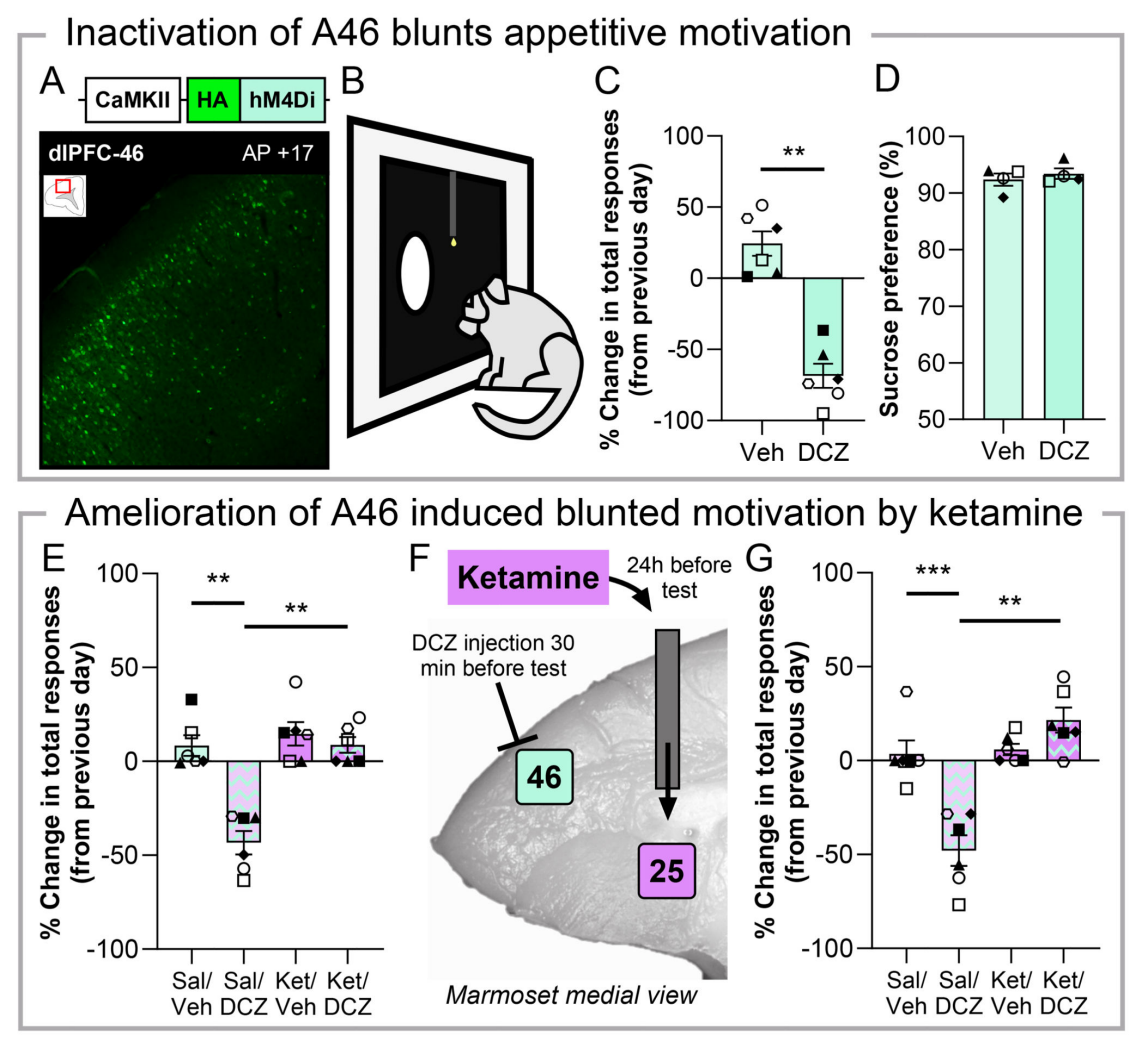
Chemogenetic inactivation of A46 blunts appetitive motivation that can be ameliorated by ketamine treatment systemically and by direct A25 infusion. (**A**) Schematic of hM4Di viral construct with photomicrograph of fused HA-tag expression in A46 with inset coronal section showing region of interest. (**B**) Marmosets respond to a touchscreen stimulus a progressively increasing number of times for reward in the progressive ratio (PR) task. (**C**) DCZ treatment decreased touchscreen responses compared to vehicle (Veh; n=6, 10μg/kg i.m. 30min before testing; paired t-test, *p*=0.0016, *d*=2.52). (**D**) Preference for 6% sucrose over water was unaffected by DCZ treatment (n=4, paired t-test, p=0.408). (**E**) Intramuscular ketamine (Ket) 24 hours prior to testing blocked the DCZ-induced reduction in responses (n=6; repeated measures ANOVA (rmANOVA) pretreat*treat interaction, F_(1,5)_=15.24, p=0.011, η^2^=0.753; post-hoc: Sal/Veh vs Sal/DCZ, p=0.001, *d*=2.62; Sal/DCZ vs Ket/DCZ, p=0.002, *d*=2.41; Sal/Veh vs Ket/Veh, p=0.508). (**F**) Schematic for assessing A25’s involvement in A46-induced motivational deficits through ketamine infusion. (**G**) Intra-A25 ketamine infusion blocked DCZ-induced reduction in responses (n=6; rmANOVA, pretreat*treat interaction, F_(1,5)_=50.34, p<0.001, η^2^=0.91; post-hoc: Sal/Veh vs Sal/DCZ, p<0.001, *d*=3.35; Sal/DCZ vs Ket/DCZ, p=0.005, *d*=1.99; Sal/Veh vs Ket/Veh, p=0.794). Data displayed as means ± SEM and individual data points with significant Sidak corrected post-hoc comparisons indicated by *.

We first studied the role of A46 in appetitive behaviors, with a deficit in this behavioral domain highly relevant to the core symptom of anhedonia in depression (20, 21). To do this we assessed a marmosets’ willingness to work for reward and their preference to consume rewarding substances. To selectively inactivate A46 neurons we infused into A46 of 6 marmosets an adeno-associated virus (AAV) containing the inhibitory chemogenetic channel hM4Di (22) under a calcium calmodulin kinase II promoter (CaMKII) (fig. S1, table S1), which primarily targets excitatory pyramidal cells (Fig. 1A). Following this, marmosets were treated with the hM4Di activator deschloroclozapine (DCZ, 10μg/kg i.m.) (23) to inactivate A46 projections. We measured the physical effort component of motivation (24) through a progressive ratio (PR) task whereby an increasing number of responses to a touchscreen stimulus are required to receive a milkshake reward (Fig. 1B, fig. S2). Performance after drug treatment was compared to the previous day’s performance to limit the impact of any inter-week variability. DCZ treatment reduced total responses when compared to vehicle treatment (30min before testing, Fig. 1C), as well as rewards received (fig. S3A). These effects occurred without significantly altering the rate of responding, although a variable decline was observed (fig. S3B), suggesting deficits in motor processes, per se, were unlikely to be the major contributor to the overall decline in total responding. In addition, this reduced motivation through hM4Di activation by DCZ was in the absence of any changes in consummatory behavior, with marmoset’s preference for sucrose unaffected during a one-hour test in the home cage following DCZ treatment (Fig. 1D), as well as the overall volume of solution consumed also unaffected (fig. S3C). These data suggest the DCZ mediated deficit in the PR task was unlikely to be related to satiety, with the maximum reward volume on the PR task (3.75mls, 25 rewards) being under 10% of the total volume consumed (~54mls) on the sucrose preference test. Prior to receiving the hM4Di-containing viral infusion in A46, we showed that DCZ has minimal off-target effects on the PR task with no behavioral measure impacted (fig. S4), indicating the specificity of the hM4Di-mediated effects.

This motivational blunting following A46 inactivation is similar to that seen following A25 overactivity (25) and is consistent with previous neuroimaging data in marmosets showing an inverse relationship between A46 and A25 activity (26). Given that anti-correlated activity between these two regions is also an indicator of neuromodulatory treatment success (12, 27) the next series of experiments determined their functional interaction with respect to treatment response. Instead of neuromodulation we explored the ability of the fast-acting antidepressant ketamine to ameliorate the A46 mediated blunting of motivation (25). Systemic treatment with ketamine (0.5mg/kg i.m., 24hrs before testing) ameliorated the blunted motivation induced by DCZ treatment, restoring both total responses (Fig. 1E) and the number of rewards received to normal levels (fig. S3D). These effects occurred with a variable but non-significant effect on response rate (fig. S3E), whilst ketamine treatment alone did not impact any behavioral variable. To determine any interaction with A25 in this treatment response, we infused A25 with ketamine directly through surgically implanted cannulae, 24 hours before testing. The A25 ketamine infusion (Fig. 1F) blocked DCZ-induced blunting of responses (0.5μg/μl; Fig. 1G) and rewards received (fig. S3F), with the response rate not significantly impacted albeit variably reduced (fig. S3G).

## Reduced motivation by pathway inactivation of A46 to A32 is blocked by ketamine in A25

**Fig. 2:**
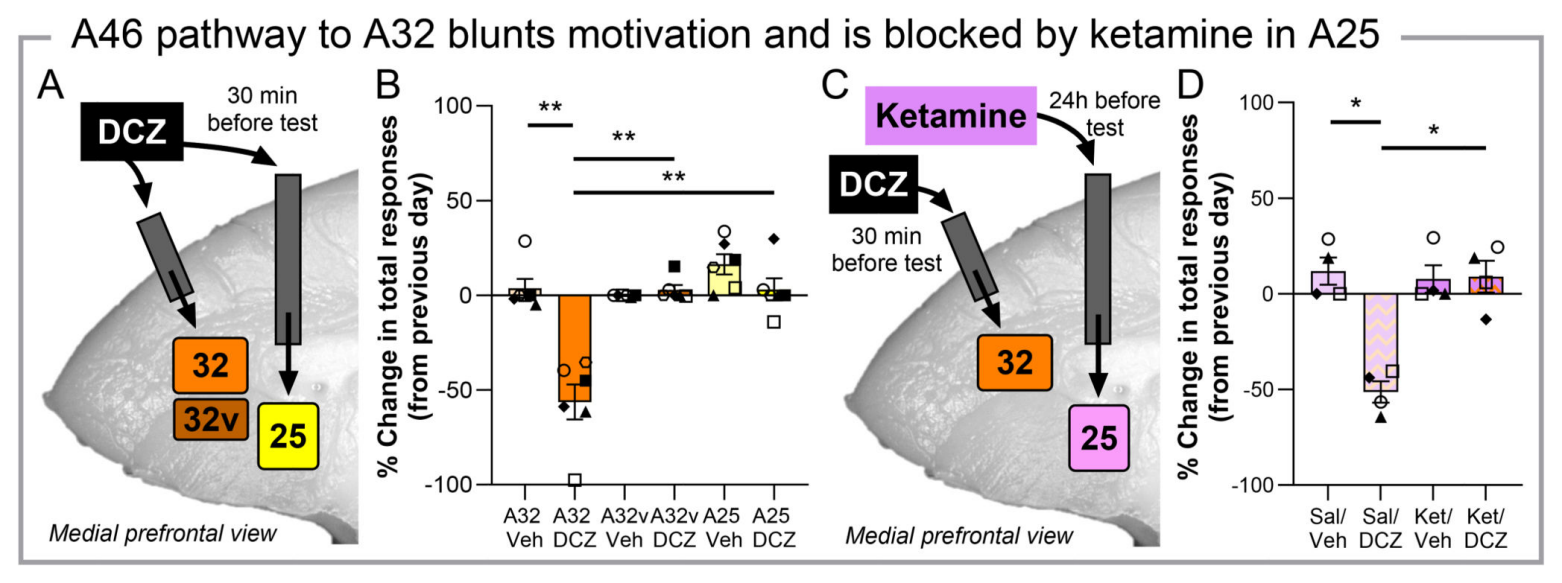
Inactivation of an A46 to A32 pathway reduces appetitive motivation with this effect blocked by a ketamine infusion in A25. (**A**) Schematic of A46 terminal pathway manipulations through DCZ infusion (100nM) in A32, A32v or A25 of the marmoset. (**B**) DCZ infusion into A32 reduced the total responses marmosets made in comparison to the previous day, with DCZ into A32v and A25 showing no effect (n=6, rmANOVA, region*treatment interaction, F_(1.73, 8.63)_=33.17, p<0.001, η^2^= 0.869; post-hoc: A32 Veh vs DCZ, p=0.001, *d*=2.77; A32 DCZ vs A32v DCZ, p=0.003, *d*=2.78; A32 DCZ vs A25 DCZ, p=0.004, *d*=2.65). (**C**) Schematic for assessing A25’s involvement in A46 to A32 pathway induced motivational deficits through A25 ketamine infusion. (**D**) Intra-A25 ketamine infusion blocked the ability of an A32 DCZ infusion from reducing responses (n=4, rmANOVA, pretreat x treat interaction, F_(1,3)_=15.31, p=0.030, η^2^= 0.836; post-hoc: Sal/Veh vs Sal/DCZ, p=0.014, *d*=2.60; Sal/DCZ vs Ket/DCZ, p=0.019, *d*=2.32; Sal/Veh vs Ket/Veh, p=0.461). Data are displayed as mean ± SEM and individual data points with significant Sidak corrected post-hoc comparisons indicated by *.

These data suggest an interaction between A46 and A25 may contribute to motivated behaviors and their dysfunction. We thus explored their direct and indirect interaction through pathway-specific manipulations induced by infusions of DCZ (100nM) into A46 neuron terminal regions via surgically implanted cannula into A25 (direct pathway) and ACC region Area 32 (A32, indirect pathway; post-mortem cannula locations can be found in fig. S5). A25 receives relatively sparse projections from A46 whilst A32 receives projections from A46 into deep cortical layers and projects extensively into A25 across all layers (28, 29). A32 has been proposed to form an indirect pathway by which A46, and dlPFC more broadly, may provide cognitive control over emotion through this serial pathway to A25 (29). Because A32 has a dorsal (A32) and ventral aspect (A32v) these were targeted separately (Fig. 2A, 30) with terminal fibres observed primarily within deeper layers, as were those in A25 (fig. S6). In the PR task, infusion of DCZ into A32 reduced the total responses made by the marmosets when compared to vehicle (n=6, Fig. 2B), as well as when compared to DCZ infusion in A32v and A25, which themselves did not differ from vehicle. A similar reduction following A32 DCZ infusion was observed for the number of rewards received, whilst the response rates were not significantly impacted though variable (fig. S7A,B).

Having established the role of the pathway from A46 to A32, but not the direct pathway from A46 to A25, in appetitive motivated behavior, we subsequently assessed its functional interaction with A25. We determined whether ketamine could ameliorate the blunting of appetitive motivation induced this time, by inactivation of the specific A46 to A32 pathway when infused into A25 (Fig. 2C). Infusion of ketamine into A25 (0.5μg/μl, 24hr pretreatment) blocked the ability of an intra-A32 DCZ infusion to reduce total responses in the PR task (n=4, Fig. 2D), with DCZ still able to induce the deficit in responses following a saline infusion into A25. Ketamine infusion in A25 did not impact responses when administered alone. These effects also extended to the number of rewards received (fig. S7C), whilst no clear impact on response rate was observed for any treatment or pretreatment (fig. S7D).

## A46 inactivation heightens marmoset’s response to ambiguous threat through a pathway to ventral A32

**Fig. 3:**
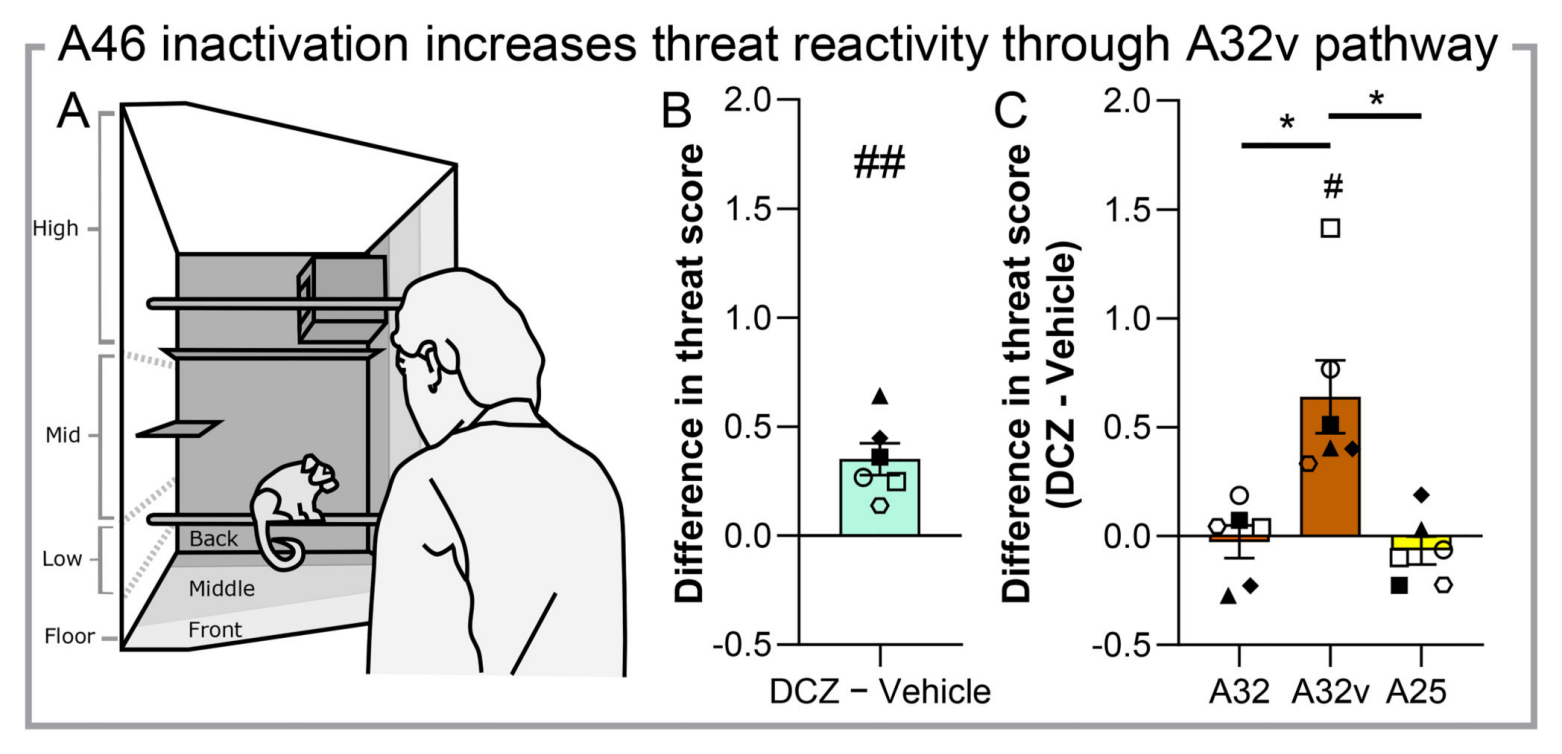
Chemogenetic inactivation of A46 heightens responsivity to ambiguous threat through projections to ventral A32. (**A**) Schematic of the human intruder paradigm setup where a novel human maintains eye contact with a marmoset for 2 minutes. A difference score between treatments is used for each marmoset to account for individual variability (n=6). (**B**) DCZ treatment (10μg/kg, i.m.) increased the threat score compared to vehicle treatment (one sample t-test vs 0, p=0.005, *d*=1.98). (**C**) DCZ infusion into A32v alone heightened the threat score when compared to vehicle infusion, and also when compared to the effects of A32 and A25 DCZ infusion (rmANOVA, region effect, F_(1.7, 8.5)_=12.52, p=0.004; post-hoc, A32 vs A32v, p=0.022, A32v vs A25, p=0.039; A32v vs 0, p=0.012, *d*=1.56). Data are displayed as means ± SEM and individual data points with significant Sidak corrected pairwise comparisons indicated by * and one sampled t-test vs hypothetical mean of 0 by #.

We next assessed whether this A46 network was involved in marmosets ‘responsivity in threatening situations. Anxiety is highly co-morbid with depression and dlPFC TMS improves co-morbid anxiety symptoms (5, 7). A25 overactivation in marmosets also heightens responsivity to threats (25). Using a novel human standing in front of the cage for 2 minutes as an ambiguous threat (Fig. 3A), we studied the effects of DCZ treatment on a range of behaviors, including the marmosets cage position and vocalisations, which are combined into a threat reactivity score (31, fig. S8). To account for individual variability, the difference in threat score between treatments for each marmoset was calculated. Systemic DCZ increased the marmosets ’threat reactivity score (Fig. 3B), an effect driven by a reduction in movement around the cage (fig. S9A). We subsequently infused DCZ into A46 neuron terminals in A32, A32v and A25. Only infusion of DCZ into A32v increased the threat score (Fig. 3C), whilst infusion into the more dorsal A32 and A25 had no effect. In this instance, the heightening of the threat score was driven more by a reduction in time spent at the front of the cage (fig. S9B).

## Evidence of functional asymmetry in A46

**Fig. 4:**
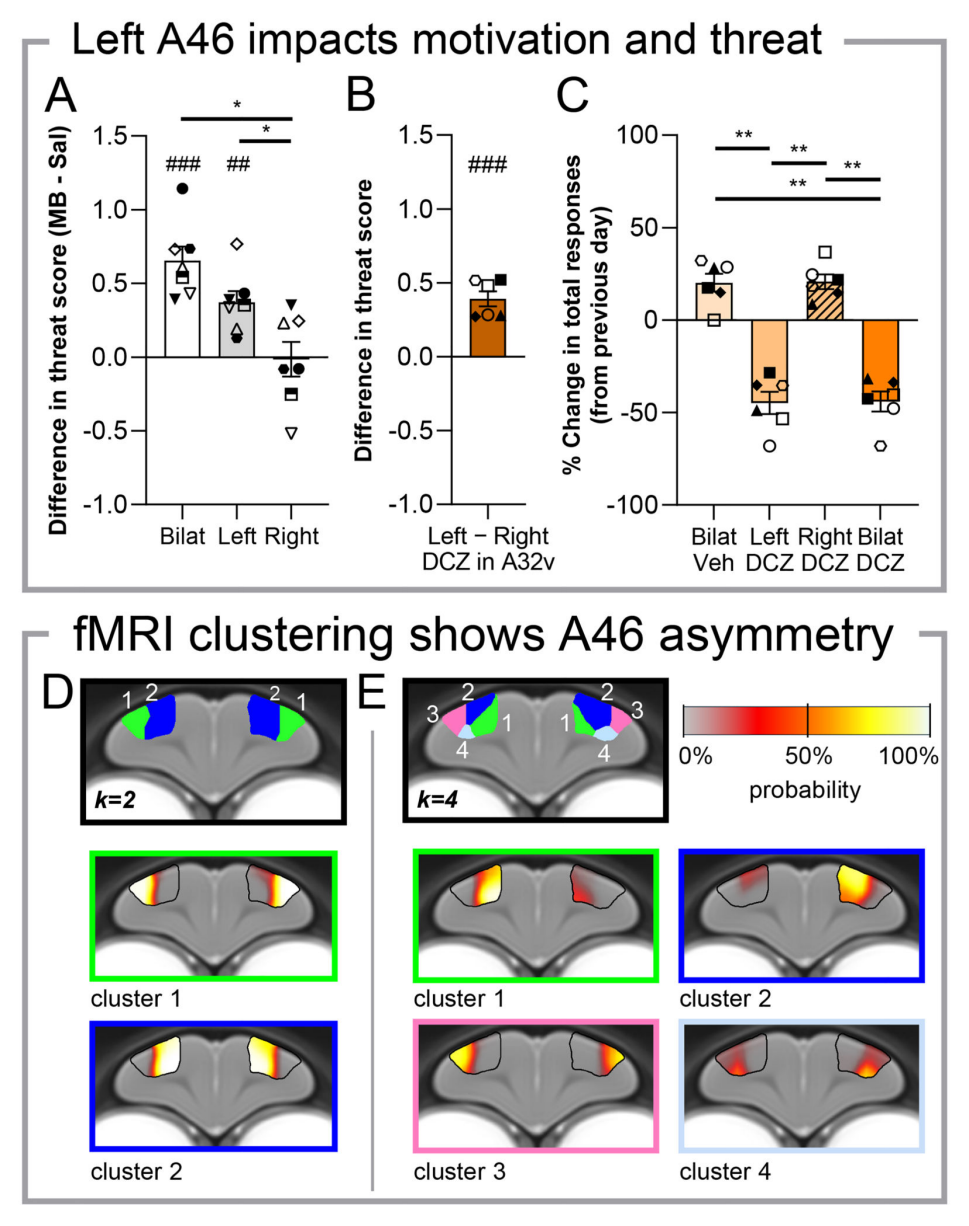
Causal evidence for functional asymmetry across A46 hemispheres through unilateral behavioral analysis and correlatory evidence from resting state functional connectivity clustering. (**A**) Bilateral and left A46 inactivation by muscimol/baclofen (MB) infusion enhanced responsivity to threat whilst right A46 inactivation had no effect (rmANOVA, region effect, F_(1.8, 10.7)_=13.5, p=0.0014; post-hoc, Bi vs Right, p=0.013; Left vs Right, p=0.041). (**B**) DCZ infusion into left A32v increased threat responsivity when compared to right A32v (one sampled t-test vs 0, p=0.008). (**C**) Left and bilateral A32 DCZ infusions reduced responses in the PR task, with right DCZ infusion having no effect (rmANOVA, region effect, F_(2.9, 14.3)_=42.8, p<0.001; post-hoc, Bilat Veh vs Left DCZ, p=0.003; Bi Veh vs DCZ, p=0.004; Left vs Right DCZ, p=0.003; Right vs Bilat DCZ, p=0.002). (**D**) K-means clustering of A46 resting state functional connectivity data in 20 subjects with k=2 indicated separate bilateral clusters that align with architectonically defined ventral (green) and dorsal A46 (blue) with high confidence, revealed by clustering probability obtained from bootstrap resampling. (**E**) K=4 clustering exposes A46 connectivity differences between pre-dominantly right (green) and left (blue) hemispheres with high confidence. Black outline indicates A46. Behavioral data displayed as means ± SEM and individual data points.

We next wanted to establish whether functional specialisation exists between hemispheres, because typical neuromodulatory strategies for treatment resistant depression are primarily unilateral (32). With a separate cohort of marmosets with intracerebral cannulae targeting A46 (n=7), we determined whether hemispheric differences were observed following unilateral and bilateral pharmacological inactivation of A46 through intracerebral infusion of GABA_A/B_ receptor agonists muscimol and baclofen (MB) directly into A46. Infusions of MB into both left and bilateral A46 increased the threat score on the human intruder test, whilst right A46 infusion had not effect (Fig. 4A, fig. S10). These findings not only replicated the chemogenetic inactivation of A46 but also indicated functional lateralization. We subsequently determined whether this extended to the established A46 pathways mediating threat and reward processes (to A32v and A32, respectively). DCZ infusion into left A32v heightened the threat score when compared to DCZ infusion into right A32v (Fig. 4B), whilst DCZ infusion into both left and bilateral A32 (but not right A32) decreased total responses when compared to vehicle on the PR task (Fig. 4C). These effects on motivation extended to the number of rewards received with the response rate unaffected (fig. S11).

To complement this data, we looked to establish whether lateral specialisation is an inherent part of the functional organisation of A46 in the marmoset brain. To do this, we studied the functional connectivity patterns of resting state fMRI data in awake marmosets from an open-access database (33) through k-means clustering analysis. When constrained to two clusters, A46 functional connectivity across all 20 subjects revealed a bilateral dorsal-ventral division consistent with its known architectonic parcellation (Fig. 4D). Increasing to four clusters revealed hemispheric lateralization: left and right dorsal A46 segments formed distinct clusters (Fig. 4E, cluster 1 [green] vs cluster 2 [blue]). These clusters showed similar ipsilateral connectivity but differed primarily in contralateral connectivity, being more pronounced in the right hemisphere. The top 30 regions showing this lateralization can be found in fig. S12, and do not include A25, A32 and A32v, suggesting that overall, broad A46 network dynamics underlie this lateralization. Cluster robustness was validated via voxelwise bootstrap resampling using half of the data each time (n=5000; Fig. 4D,E, lower section).

## Discussion

A46 within the dlPFC forms a functional network with A32 and A25 for regulating positive and negative emotion-related processes in marmosets. Inactivation of A46 blunts appetitive motivation and heightens responsivity to uncertain threat, two behavioral phenotypes highly relevant to our understanding of anhedonia and anxiety in depression. This functional network utilises separate A46 projections to dorsal and ventral A32, to regulate these emotion-related behaviors. Additionally, we highlight the important role of A25 within this network to ameliorate A46 inactivation-induced appetitive motivational deficits, acting as a putative site of action for the fast-acting antidepressant, ketamine. Moreover, we provide evidence of functional asymmetry as the global and pathway-specific effects of A46 inactivation are left, but not right dependent. Together these effects provide functional evidence for the hypothesised interaction between these regions (29, 34).

The function of A46, and the dlPFC more generally, has been associated with maintaining an online representation of information that can be utilised to regulate many different behaviors and is highly sensitive to stress (35–37). Here we demonstrate that marmoset A46 is necessary for maintaining an escalating amount of effort to acquire rewards whilst not directly being involved in the hedonic value of rewarding substances themselves. These findings are in agreement with the correlatory evidence that dlPFC activity in humans represents both cognitive and physical effort during decision making (38) and that dlPFC neurons in macaque monkeys encode motivational variables with increasing activity during lower value trials; the latter hypothesised to increase tolerance for periods of reduced reward (39). Moreover, they appear consistent with the blunting of cognitive effort humans were willing to exert following dlPFC dysfunction induced by non-invasive stimulation (40). Thus, the current deficit may be due to impaired integration of effort and reward value in the PR task resulting in an intolerance for the escalating effort cost for each reward. That said, the PR data shown here cannot directly parse the discussed motivational deficit from an increased intolerance for the delay in reward or enhanced fatigue.

The dlPFC, and A46 by extension, has also been associated with the cognitive regulation of negative emotional responses, where it acts to appraise or re-appraise uncertain threat (41) or suppress episodic recall and affective content (42). Reduction of this top-down cognitive control from A46, induced by either pharmacological or chemogenetic inactivation, likely impaired marmoset’s ability to appropriately appraise the uncertain threat context or adequately suppress threat-related circuits from altering their behavioral strategy, resulting in intolerance to the uncertain threat of the human intruder and a heightened response (31).

That A46 inactivation induced both anhedonia-like and anxiety-like effects raises the question as to whether these two phenotypes are related? Indeed, the dlPFC is active during approach-avoidance tasks in humans involving conflict between reward and threat (43) with decreased activity correlated with anhedonia in individuals with depression (44). Nonetheless, our current findings reveal these phenotypes as doubly dissociable at the level of downstream pathways highlighting their independence; with the A46 pathway to A32 regulating appetitive motivation, whilst the A32v pathway regulates responsivity to threats. A32v is a relatively small and understudied structure in marmosets, with prior data suggesting the involvement of dorsal A32 in regulating negative affect (45, 46), thus further characterization of this region will be required in the future. In macaque monkeys a similar ventral, but not dorsal, region of A32 was observed to be selectively involved in generating avoidant behavior, an effect which was ameliorated by anxiolytic drug treatment (47). In humans and macaques A32 has been identified as a connector hub and our findings support the hypothesis that A32 is positioned to integrate frontal cortex across distinct functional modalities, including the mediation of cognitive control over emotion (48). Afferents from A46 to A32 have been identified as predominantly feedforward with neurons in the superficial layers of A46 projecting primarily to the deep layers of A32 (29). Comparative analysis of A32 terminals on excitatory and inhibitory neurons of all layers of A25 suggests that A32 may have a primarily inhibitory impact on A25, thereby mediating the link between the cognitive control functions of A46 with emotion processing in A25.

The exact circuits underpinning treatment efficacy of dlPFC neuromodulation are still being established despite their clinical use for depression for over 15 years (5, 6). However, repetitive (r)TMS treatment efficacy across dlPFC sites is primarily observed for those areas anti-correlated with scACC activity, including A25 (27). Here we demonstrate that direct ketamine infusion into A25 produced long-term changes that blocked the appetitive motivational deficits arising from A46 inactivation, an interaction likely mediated via A32, consistent with this region’s major inter-connectivity with both A46 and A25 in macaques (29) and aligning with marmoset connectivity data (49). Marmoset and macaque studies have reported, respectively, reduced activity or functional connectivity in A46 following A25 overactivation further illustrating this functional interaction (26, 50). The underlying mechanism by which ketamine within A25 has its ameliorative effects is currently unclear, although ketamine can drive long term spinogenesis in ventromedial prefrontal regions in rodents (51). Given that overactivation of A25 can mediate different components of anhedonia-like behaviour in marmosets and produce an overall aversive affective state (25, 34), the ability of ketamine to alter A25’s downstream response to A46 inactivation may be the mechanism of this ameliorative process. While this would be consistent with A25 acting as a downstream effector of the hierarchical pathway from A46, via A32, nevertheless it cannot be ruled out that A25 acts instead as a more general modulatory hub. Only future development of trans-synaptic technologies will be able to functionally differentiate these possibilities.

Asymmetry has been observed not only in the functions of human dlPFC but also their dysfunction in disorders such as depression. This has resulted in neuromodulatory strategies being hemisphere dependent, with left sided rTMS the more established treatment option (5, 6). Here we provide evidence for functional asymmetry within A46 of the marmoset dlPFC, shown both by pharmacological manipulation and chemogenetics as well as functional imaging in a larger independent cohort of marmosets. We have shown that reactivity to uncertain threat and appetitive motivation is governed selectively by the left hemisphere. Consistently, recent evidence implicates appetitive motivation in humans with left dlPFC (52) and improvements in effortful motivation in depressed individuals by left dlPFC rTMS (53). Whilst we observed no particular behavioral process dependent upon right A46 function here, rs-fMRI connectivity patterns nevertheless revealed overall greater cross-hemisphere connectivity in the right hemisphere; although the functional relevance of this remains to be determined. However, it is of interest that right dlPFC in humans is differentially activated during inhibitory control mechanisms, including that for emotional memories (4).

In conclusion, this study has identified a functional network for A46 within the left hemisphere that is involved in the regulation of positive and negative emotion-related behaviors in marmosets and provides insight into the mechanisms by which ketamine may act to ameliorate motivational anhedonia. These data provide a window onto the neurocircuitry within prefrontal and cingulate cortices that regulate emotion-related behaviors, providing a functional framework for understanding not only how dysfunction in A46, and the dlPFC more generally, can impact symptom-related behaviors in depression and anxiety but also how various treatment strategies may have their therapeutic action.

## Supplementary Material

The source data can be found at Reference 54. Specifically the link - https://doi.org/10.17632/7p2h8pktdk.1

Figs S1-S12, Tables S1-S2

## Data Availability

All data and connectivity analysis code are available in the main text, supplementary materials or deposited at https://doi.org/10.17632/7p2h8pktdk.1 (54). Raw resting state fMRI connectivity data can be found at https://www.marmosetbrainconnectome.org/download.html. Viral construct containing hM4Di was acquired from VectorBuilder (vector information found here - https://en.vectorbuilder.com/vector/VB221010-1182nft.html).
