# Supplementary material for: Dysfunction in primate dorsolateral prefrontal area 46 affects motivation and anxiety
Source: Science. Author manuscript; Available in PMC 2025 Oct 2. (PMC7618107; doi:10.1126/science.adx4142)
Supplement: Figs S1-S12, Tables S1-S2 [file EMS207967-supplement-Figs_S1_S12__Tables_S1_S2.pdf]

Science
AAAS

Supplementary Materials for

Dysfunction in primate dorsolateral prefrontal area 46 affects motivation and anxiety

Christian M Wood[1]*†, Rana Banai-Tizkar[1]*†‡, Martina Fort[1], Xinhu Zhang[1], Kevin G Mulvihill[1], Naixuan Liao[1], Gemma J Cockcroft[1], Lauren B McIver[1], Stephen J Sawiak[1], Angela C Roberts[1,2]*

Corresponding authors: cmw84@cam.ac.uk; rb869@cantab.ac.uk; acr4@cam.ac.uk

The PDF file includes:

Materials and Methods
Figs. S1 to S12
Tables S1 to S2
References (55-65)

Other Supplementary Materials for this manuscript include the following:

Data S1

Materials and Methods

Subjects

Fifteen experimentally naïve common marmosets participated in these studies (Callithrix jacchus, 7 females; see Table S1 for a breakdown of subjects). They were bred on-site at the University of Cambridge Marmoset Breeding Colony and housed in male/female pairs (males were vasectomised) on a 12-hour light-dark schedule (7am lights on). Marmosets were housed in a tall environmentally enriched cage (280 x 120 x 98 cm), receiving a varied diet of MP.E1 primate pellets (Special Diet Services, UK), carrots, fruit, rusk, eggs and bread. During the testing phase of the chemogenetic study, marmosets' access to water was restricted on weekdays to provide stable motivation across progressive ratio test sessions, with ad libitum water available across weekends and holidays. All procedures were carried out in accordance with the UK Animals (Scientific Procedures) Act 1986 and the University of Cambridge Animal Welfare and Ethical Review Body.

Surgical procedures

For the chemogenetics study, marmosets received bilateral infusion of an adeno-associated virus into Area 46. Following initial testing and systemic drug treatment, they then received surgery to implant chronic indwelling cannulae targeting Area 32 and Area 25 – identical to that performed previously in marmosets (*25, 26*). For the pharmacological study, marmosets received bilateral indwelling cannulae targeting Area 46 alone.

Prior to each surgery, marmosets were premedicated with ketamine hydrochloride (10mg in 0.1ml i.m., Vetalar, Amersham Biosciences and Upjohn), along with the nonsteroidal anti-inflammatory analgesic meloxicam (1.5mg in 0.075ml s.c., Metacam, Boehringer Ingelheim). Anaesthesia was induced via a face mask (4% isoflurane, 0.7L/min O_2), and maintained following intubation (2.0–2.5% isoflurane, 0.3-0.4 L/min O_2). Respiration, heart rate (HR), O_2 saturation, and CO_2 blood levels were continuously monitored using a pulse oximeter capnograph (Microcap Hand-held Capnograph, Oridion Capnography), as well as body temperature (TES-1319 K-type digital thermometer).

For targeting distinct brain regions, marmosets were placed in a stereotaxic frame (David Kopf). Anteroposterior co-ordinates for each surgery were adjusted in situ following two cortical depth checks, the first within the prefrontal cortex at +17.5 anteroposterior (AP), -1.5 lateromedial (LM) at a range of 5.8-6.8mm (*55*) and the second within the ventromedial prefrontal cortex, +14.0 AP, -1.0 LM at a range of 8.9-9.3mm (*56*).

At the end of surgery, marmosets received dexamethasone (sodium phosphate, 0.18ml of 3.8mg/ml i.m., Aspen Pharma) to limit central inflammation, as well as close post-operative monitoring until fully recovered. Meloxicam (0.15mg in 0.1ml oral, Metacam, Boehringer Ingelheim), an analgesic, was administered for three consecutive days post-operatively.

<u>Surgical infusion of adeno-associated virus containing chemogenetic construct into A46</u>

Surgical infusion under anaesthesia took place similar to the protocol described previously (*57*). Briefly, marmosets received a bilateral infusion of an adeno-associated virus (AAV8-CaMKII-

HA-hM4Di, 1µl of 1 x 10^{10} vg/µl, Vectorbuilder) into A46 (+17.5 AP, ±3.3 LM, -0.8 DV from cortical surface) at a rate of 0.1µl/min for 10 minutes, with a 10-minute wait time afterwards to allow for diffusion. It should be noted that the CaMKII promoter primarily targets excitatory neurons but is not exclusive, with evidence in marmosets suggesting some simultaneous off target expression in inhibitory neurons (*58*). Infusions took place through a gas tight 10µl Hamilton syringe connected to a surgical injector (31GA, C316I/SPC 25mm long with 45° bevelled edge, Plastics One) mounted on the stereotaxic frame (1776-P1, David Kopf). Following surgical infusion, the skin incision was sutured together (3-0 Vicryl, Ethicon), they were then recovered from surgery and subsequently returned to their home cage.

Implantation of chronic indwelling intracerebral cannulae

Implantation of cannulae under anaesthesia followed a similar protocol to that reported in detail previously (*59*). For the chemogenetic study bilateral double cannulae (C235G-1.4/SPC cut 7mm below pedestal, Plastics One) were targeted for Area 32 and Area 25 through small holes drilled into the skull at specific co-ordinates (Area 32, +17.5 AP, ±0.7 LM; Area 25, +14.0 AP, ±0.7 LM), with insertion into the cortex so cannula rest above the region of interest (Area 32/32v: -2.5mm from cortical surface at 23° angled anterior from vertical; Area 25: -2/3 of cortical depth vertical from cortical surface). For the pharmacological study, single cannulae were implanted bilaterally, targeting A46 on each side (C316G/SPC cut 3.5mm below pedestal, +17.5 AP, ±3.2 LM, -0.3mm below cortical surface). Cannulae were secured through dental acrylic (Paladur) applied to the top of the skull containing skull screws at 4 secure points. Dummies and caps were placed into the cannulae to maintain their patency (double cannula, C235D-1.4/SPC; single cannula, C316DC/SPC; Plastics One). Following this, the skin incision was sutured together and then marmosets recovered from surgery and subsequently returned to their home cage. A weekly cleaning of the implant was performed using 70% ethanol (v/v), and sterile dummy cannulae and caps were replaced.

Drug treatments

Before any drug treatment, marmosets were habituated to the handling procedure for either central or peripheral drug administration, with marmosets held gently by an assistant familiar to them.

Systemic injections

DCZ (10 µg/kg) was dissolved in 5% DMSO saline for 0.1ml intramuscular injections in the quadricep, with alternating legs used for subsequent injections. A pretreatment time of 30 minutes was used for systemic DCZ injections, as has been used previously (*57, 60*). Validation of no off-target effects with DREADD actuators in marmosets was provided previously (*57*) for uncertain threat in the form of an unfamiliar human, and is shown here for appetitive motivated behaviours in fig. S4. Intervals between DCZ/Vehicle injections were typically 1 week. Racemic ketamine hydrochloride (Tocris, UK) was diluted in 0.9% sterile saline with a final administered dose of 0.5mg/kg, 24 hours prior to behavioural testing. Ketamine/saline injections were separated by 3 weeks to account for the longer-term effects of ketamine that subside between 7 and 21 days post-treatment (*25*, *57*).

Intracerebral microinfusions

Caps and dummies were removed from the guide cannulae, and the site was cleaned with a 70% isopropyl alcohol wipe (Alcotip). Sterile injectors (double cannula, C235I-1.4/SPC; single cannula, C316I/SPC; Bilaney Consultants, UK) connected to a 10-µl gas tight syringe pump through PTFE tubing were inserted into the guide. The length of the injector was determined by the cortical depth and the placement of the guide cannula during surgery. Injectors for A32v were specifically 1mm longer than those for A32, enabling clear separation between manipulations on both dorsoventral and anteroposterior axes owing to the angled nature of the guide cannula. Bilateral infusions were carried out for 2 min at a rate of 0.5 µl/min for DCZ (100nM diluted in sterile 1% DMSO saline; *57*, *61*), ketamine hydrochloride (0.5µg/ul diluted in 0.9% sterile saline) and their appropriate vehicles. The cocktail of $GABA_{A/B}$ receptor agonists muscimol and baclofen (0.1mM muscimol/1.0mM baclofen diluted in 0.9% saline, 'MB'; Sigma Aldrich, UK) were infused for 2 minutes at a rate of 0.25 µl/min (*56*). Sterile dummies and caps were then replaced, and the marmoset was returned to its homecage for the appropriate pretreatment period (30 minutes, DCZ; 25 minutes, MB). For unilateral manipulations, drugs were administered to the specified hemisphere with the other hemisphere receiving the appropriate vehicle. The interval between DCZ/Vehicle infusions were typically 1 week, whilst ketamine/saline infusions were separated by 3 weeks to enable the longer-term effects of ketamine to subside (*25, 57*). The interval between infusions of MB and its corresponding saline were 2 weeks. Drug infusion order was counterbalanced across subjects.

Behavioral testing

Before any behavioral testing, marmosets were trained to enter a transparent Perspex box (240 mm by 230 mm by 200 mm) in which they were transported to the behavioural touchscreen testing apparatus for the progressive ratio paradigm. Both the human intruder and sucrose preference tests were conducted within the top right quadrant of the home cage. Across all behavioural paradigms, within-subject controls were used throughout.

<u>Progressive Ratio paradigm</u>

This paradigm assessed the amount of physical effort a marmoset would expend to receive a reward (fig. S2) and was conducted according to procedures used previously (*25,62*). After training on the touchscreen, marmosets pressed a circular white stimulus to receive a reward (5 seconds of milkshake, ~10% w/v banana Nesquik in whole milk, 0.15mls) from a spout central to the screen, and was accompanied by 5 seconds of birdsong (80dB). Maximum rewards received across experimentation were 25, totalling ~3.75mls of reward. The number of required responses increased after each reward, such that the reward point at which marmosets stopped responding was termed the breakpoint. The required number of responses increased by 1 for the first 8 trials, with this increment doubling every 8 rewards to a maximum incremental increase of 8 (*63*). Sessions concluded after 30 minutes or two minutes of inactivity. Measures utilised in this test include the total number of responses a marmoset made during the test session, the reward number received after which marmosets stopped responding – 'breakpoint' and the response rate (time between first and last touches in the session ÷ total responses). To account for inter week and subject variability, mock procedures were carried out the day immediately prior to drug manipulation (all handling processes minus the drug administration procedure) with a percentage change in behavioural variables compared between drug manipulations and mock procedure days (the day previous) using the formula: (variable on manipulation day – variable on mock day)/variable on mock day x 100. Marmoset's performance across the study remained

stable, with the average total responses across a week during the early, middle and late phases of the study illustrated in Table S2.

Human Intruder test

Testing was conducted similarly to that previously described (*57*). Marmosets were separated from their cage mate into the upper-right quadrant of their home cage 8 minutes before the end of the pretreatment time. After this, an unfamiliar human intruder entered the room and stood 40 cm from the cage, maintaining eye contact throughout the 2 minute test period. The intruder was a researcher wearing a realistic human mask (Masks Direct) unfamiliar to the marmoset and wearing familiar scrubs. The order of masks and drug treatments were counterbalanced across marmosets, with at least 2 weeks between each test. Behaviour was recorded using a camera (GoPro Hero5), and a microphone was used to record vocalizations (Sennheiser MKE 400). Behavioural scoring was conducted by individuals blind to treatment and subject with JWatcher software, measuring the time spent in different zones (front, middle, back for the horizontal plane; floor, low, middle, high, and top of the nest box for the vertical plane, as shown in Fig. 3A). Sound recordings were converted to spectrograms in Syrinx software to count specific vocalisations (tsik, egg, tsik-egg, and tse-egg). Measures such as the percent time spent at the front and back, as well as the average height of the subject, locomotion, head and body bobs, and vocalisations all contributed to the exploratory factor analysis (EFA) score — a composite anxiety-like behaviour score produced from analysis of the human intruder test behaviour of 171 marmosets within the Cambridge marmoset facility by Quah et al (*31*). To produce this composite score, specific weightings for these individual measures are outlined in fig. S8. To account for intersubject variability, a difference score between vehicle and drug treatments (systemic injections or specific regions for drug infusion) was calculated for the EFA-calculated threat score and each individual behavioural measure.

Sucrose preference test

Testing was conducted identically to previous reports (*62*). Briefly, marmosets were initially habituated to the 6% sucrose solution (w/v in tap water; Sigma-Aldrich) in two bottles in the top right quadrant of the home cage for 48 hours. Subsequently, marmosets were separated from their cage mate into the upper right quadrant of the home cage and usual water bottle removed. They were then presented with identical plastic bottles, one containing tap water, the other the 6% sucrose solution for one hour. Once marmosets reached a stable sucrose preference (>90% for 2 sessions), experimental manipulations took place, whereby consumption of water and sucrose were calculated every 30 minutes through measuring the weight change in the bottles. Sucrose preference was calculated by creating a percentage of the sucrose consumption from the total consumption (sucrose and water).

Functional connectivity analysis from online marmoset resting state resource

The resting-state functional magnetic resonance imaging (rs-fMRI) data analysed here is from an open-access online database (*33*). We used pre-processed data from 20 subjects (subjects 6-8, 11-12, 14-16, 18-20, 22-28 and 30-31) comprising a total of 154 rs-fMRI recordings (6-8 per subject). We aligned these to a previously published marmoset atlas developed at the University of Cambridge (*64*). To assess connectivity, we concatenated each subject's recordings before computing Pearson's correlation coefficient (r) between the functional activity timeseries of each voxel in A46 (in either hemisphere) and all other regions of interest (ROIs) in the whole brain

except A46 itself (ROIs = 299). The timeseries for each ROI was the arithmetic mean of all voxels within it. The resulting connectivity matrix of *r* values was then converted to Student's *t*-values as

$$t = r\sqrt{\frac{n-2}{1-r^2}}$$

where *n* represents the number of time points in the concatenated series. Group level statistics were calculated across all 20 subjects using a *t*-test for each voxel-ROI pair.

To identify functional activity clusters within A46, we applied k-means clustering to the group level connectivity matrix (MATLAB 2024a, Mathworks Inc.) for k=2 and k=4. To find genuine network differences rather than proximity effects, ROIs were considered not as left or right but as ipsi- or contra-lateral to each voxel under consideration. In this way, considering a single connection as an example, a voxel in left A46 strongly connected to left A8 but weakly connected to right A8 would match well with a voxel in right A46 which happened to be strongly connected to right A8 but weakly connected to left A8.

Confidence in the clustering results was calculated by bootstrap resampling ($n = 5{,}000$) using half of the subjects and counting how often a voxel was in the same cluster from the full dataset. Cluster assignments were matched using the Hungarian algorithm (*65*).

Statistical analyses

All figures were produced within GraphPad Prism 10 or MATLAB (2024a, Mathworks), with statistical analysis within IBM SPSS statistics (v29, IBM, USA). Effects of systemic injection of DCZ or vehicle were analysed by paired t-test for progressive ratio and sucrose preference data. Within subject repeated measures analysis of variance (rmANOVA) were conducted for all ketamine-related data sets on the PR task with two factors, pretreatment (ketamine or saline) and treatment (vehicle or DCZ). For pathway analysis in the PR task, a similar within subject rmANOVA was conducted with two factors, region (A32, A32v and A25) and treatment (vehicle and DCZ). For analysis of asymmetry in the PR task, a within subject rmANOVA was conducted with region as a factor (vehicle, left DCZ, right DCZ and bilateral DCZ). For all human intruder comparisons, difference scores were produced for the overall threat response score and individual measures to account for individual variability. Data were analysed by a one-sampled t-test vs a hypothetical mean of 0 to show drug effects or comparison between unilateral manipulations, whilst comparisons between groups were conducted by a one-way rmANOVA with region as the within subject factor (e.g. A32, A32v and A25). For all ANOVA, F, p, and partial eta-squared (η^2) values are reported throughout where appropriate, with the Huynh-Feldt correction used when sphericity was violated. Appropriate post-hoc comparisons used Šidák correction with p value and Cohen's *d* reported where appropriate. Statistical significance is indicated by * for $P < 0.05$, ** for $P < 0.01$, and *** for $P < 0.001$ in all figures.

Post-mortem assessment of cannulae placement

Marmosets were premedicated with ketamine hydrochloride (10 mg, i.m.) and subsequently euthanized with sodium pentobarbital (200 mg, iv; Dolethal). Marmosets were then transcardially perfused with ice-cold 0.1 M phosphate-buffered saline (PBS; Sigma-Aldrich) followed by 4% formaldehyde solution (VWR International). The brain was removed and placed

in 4% formaldehyde solution overnight, then 0.01 M PBS-azide for 48 hours, and lastly 30% sucrose for 72 hours. The brain was sectioned using a freezing microtome (40 µm) in five series. One series was mounted on gelatin-coated slides and stained with cresyl violet to verify the accurate placement of the cannula. For the marmosets from the chemogenetic study, one series was used to assess hemagglutinin (HA)–tag staining, identical to that reported previously (*57*). For HA-tag staining, sections were washed in 0.01 M PBS–Triton X-100 (PBS-T, 0.3% (v/v); Sigma-Aldrich, UK) three times for 10 min, blocked for 2 hours with 3% normal goat serum/1% bovine serum albumin solution (w/v in 0.01 M PBS-T) before being incubated overnight with primary antibody (1:400 anti HA-tag primary Rabbit antibody diluted in 3% NGS 0.01M PBS-T; Cell Signaling Technology, catalog no. 3724, RRID:AB_1549585). The following day, sections were washed in PBS-T and then incubated for 2 hours with the secondary antibody (1:1000 goat anti-rabbit Alexa 488 diluted in PBS-T; Abcam, catalog no. ab150077, RRID:AB_2630356), after which sections were washed in 0.01 M PBS, mounted onto Superfrost slides using VECTASHIELD Vibrance antifade mountant, and coverslipped.

Visualisation and photography of the sections were conducted using a M205FA stereo microscope (Leica, UK). Histological assessment confirmed the expression of the HA-tag fused hM4Di in A46 (fig. S1) and successful placement of cannula into A46 (fig. S10), A32 and A25 (fig. S5).

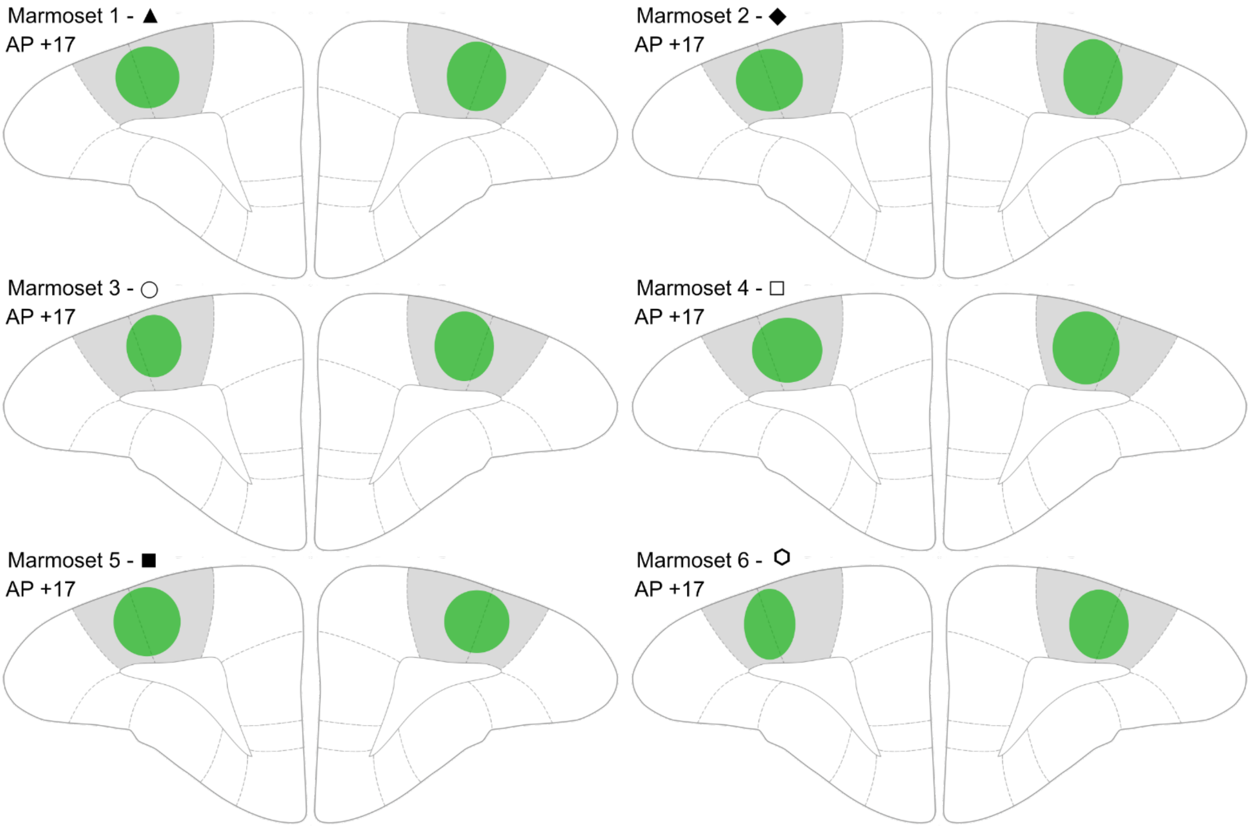

Fig. S1. Schematic representation of HA-tag fused hM4Di expression in A46 of marmosets that received targeted AAV surgical infusion. Individual marmosets and their representative data symbol are provided, with light grey shading for A46 based on the marmoset atlas including both dorsal and ventral A46 subregions (*30*). Green ellipses indicate the extent of viral expression location within each marmoset across both hemispheres. Post-mortem HA-tag staining was observed 22 months (Marmoset 1-4) or 15 months (Marmoset 5-6) after viral infusion surgery.

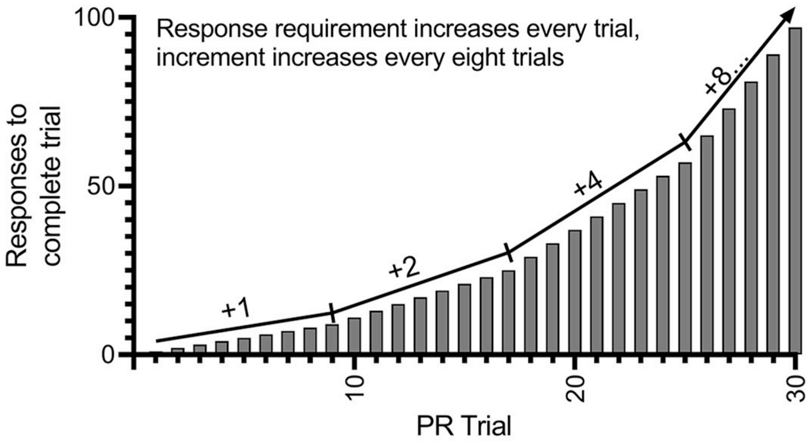

Fig. S2. Progressive ratio schedule indicating response requirement per reward. In this touchscreen task, marmosets must press a stimulus to receive a milkshake reward, with the number of presses (responses) steadily increasing after each reward received. Initially, the incremental increase of required responses is +1 (i.e. 1, 2, 3 4 responses), which doubles every 8 rewards (+2 after 8 rewards, +4 after 16 rewards) until a maximum of +8. This schedule was taken from Pryce et al (*63*) and has been used previously to study the effect of alterations in subcallosal cingulate area 25 activity in marmosets (25*, 62*).

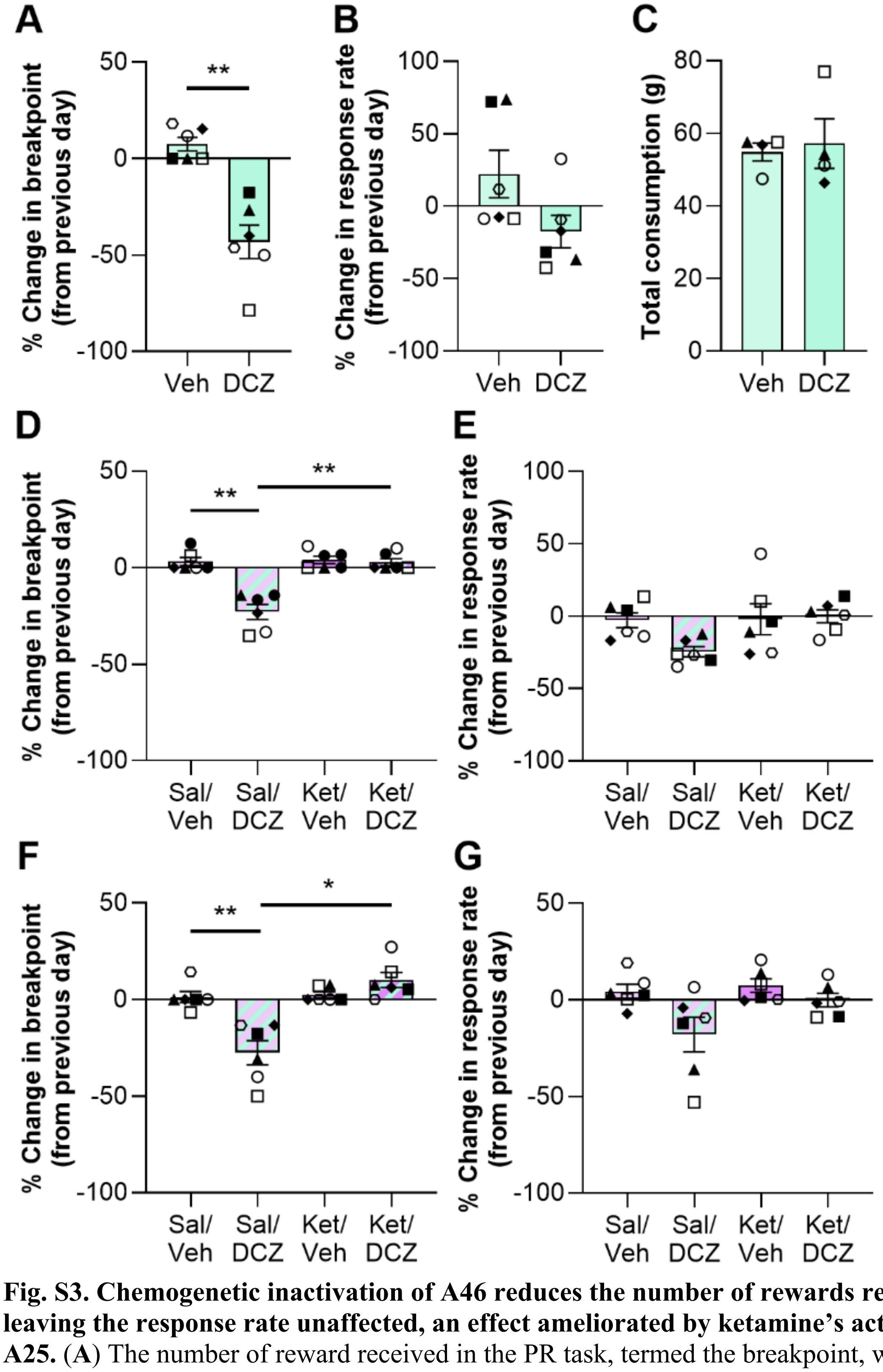

Fig. S3. Chemogenetic inactivation of A46 reduces the number of rewards received whilst leaving the response rate unaffected, an effect ameliorated by ketamine's action within A25. (**A**) The number of reward received in the PR task, termed the breakpoint, was reduced in marmosets that received DCZ treatment (n=6, 10ug/kg; paired t-test vs Veh, p=0.033, *d* =2.15). (**B**) Marmosets rate of pressing the touchscreen stimulus in the progressive ratio task was

unaffected by DCZ treatment (n=6, paired t-test vs Veh, p=0.157). (**C**) In the sucrose preference test, DCZ treatment did not influence the total amount of solution consumed (p=0.738). (**D**) This DCZ-induced reduction in breakpoint was blocked by systemic ketamine treatment (0.5mg/kg, 24hrs prior), with ketamine alone having no effect (rmANOVA, pretreatment*treatment interaction, $F_{(1,5)}$=17.241, p=0.009, η^2=0.775; post-hoc: Sal/Veh vs Sal/DCZ, p=0.002, *d*=2.4; Sal/DCZ vs Ket/DCZ p=0.003, *d* =2.3; Ket/Veh vs Sal/Veh p=0.769). (**E**) The response rate during each session was unaffected by either ketamine pretreatment or DCZ treatment (rmANOVA, pretreatment*treatment interaction, $F_{(1,5)}$=5.11, p=0.073, η^2=0.505). (**F**) Infusion of ketamine into A25 blocked the ability of systemic DCZ treatment to reduce the breakpoint (n=6, rmANOVA, pretreatment*treatment interaction, $F_{(1,5)}$=23.09, p=0.005, η^2=0.822; Sidak corrected post-hoc: Sal/Veh vs Sal/DCZ, p=0.002, *d*=2.42; Sal/DCZ vs Ket/DCZ, p=0.011, *d*=1.61; Sal/Veh vs Ket/Veh, p=0.768). (**G**) The rate of responses was unaffected by neither ketamine or DCZ treatment (rmANOVA, pretreatment*treatment interaction, $F_{(1,5)}$=3.712, p=0.112). Data are displayed as means ± SEM with individual data points.

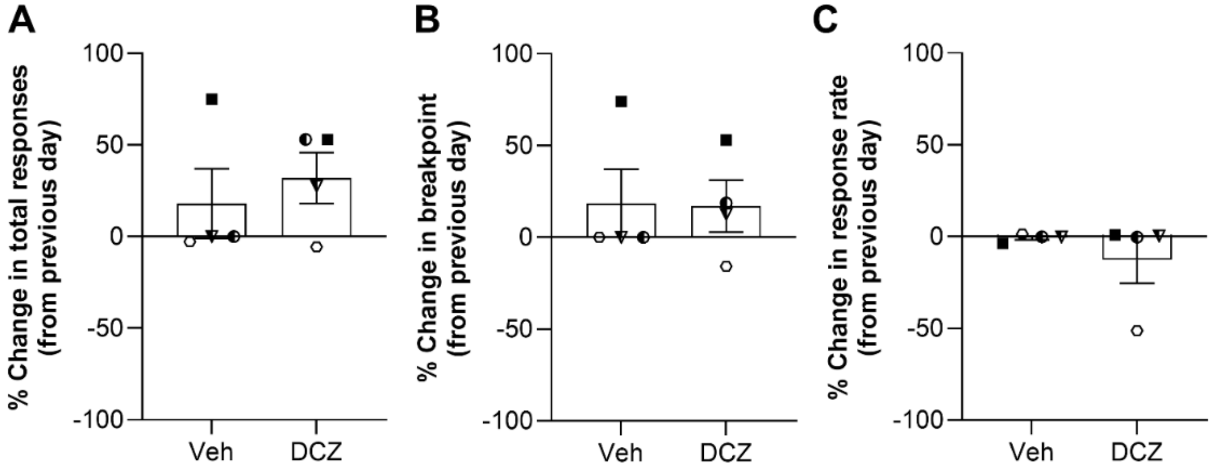

Fig. S4. Deschloroclozapine treatment does not impact behavioural measures in the progressive ratio task prior to chemogenetic viral infusion. (**A**) In marmosets prior to chemogenetic virus surgery, the total number of responses in the PR task was unaffected by DCZ treatment (10ug/kg) when compared to vehicle (n=4, paired t-test, p=0.461). (**B**) Similarly, DCZ treatment did not affect the number of rewards received when compared to vehicle (paired t-test, p=0.897). (**C**) Finally, the rate of responding in the PR task was not affected by DCZ treatment when compared to vehicle (paired t-test, p=0.447). Data are displayed as means ± SEM with individual data points.

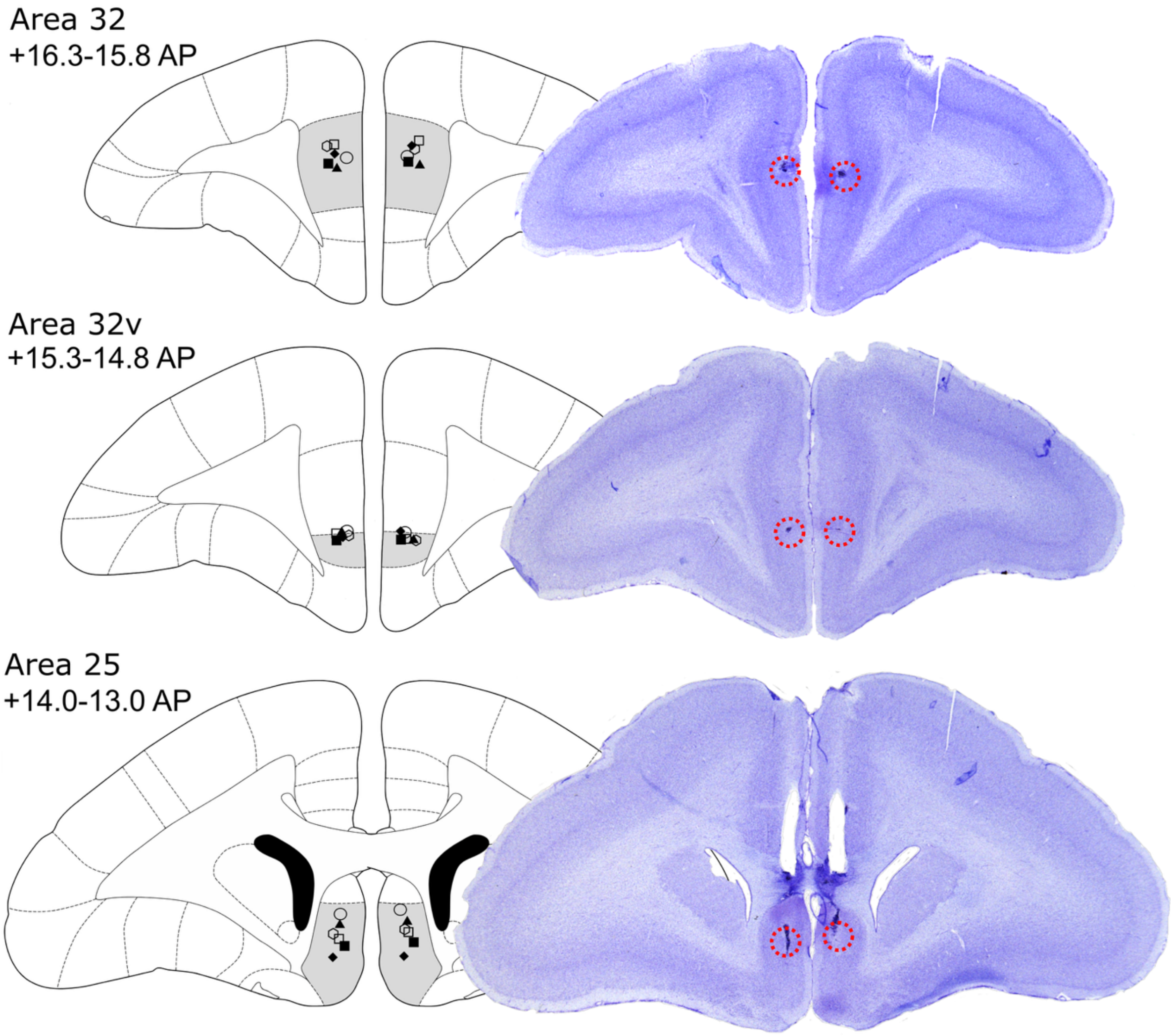

Fig. S5. Histological verification of infusion targeting to Area 32, Area 32v and Area 25. Schematic coronal sections for Area 32 (top left), Area 32v (middle left) and Area 25 (bottom left) represent the anteroposterior co-ordinate locations of infusions with light grey shading indicating each region of interest. Subject-specific symbols indicate individual localisation within each region for each marmoset. Representative cresyl sections on the right are taken from one subject (open circle) with each cannula location indicated by a red dashed circle.

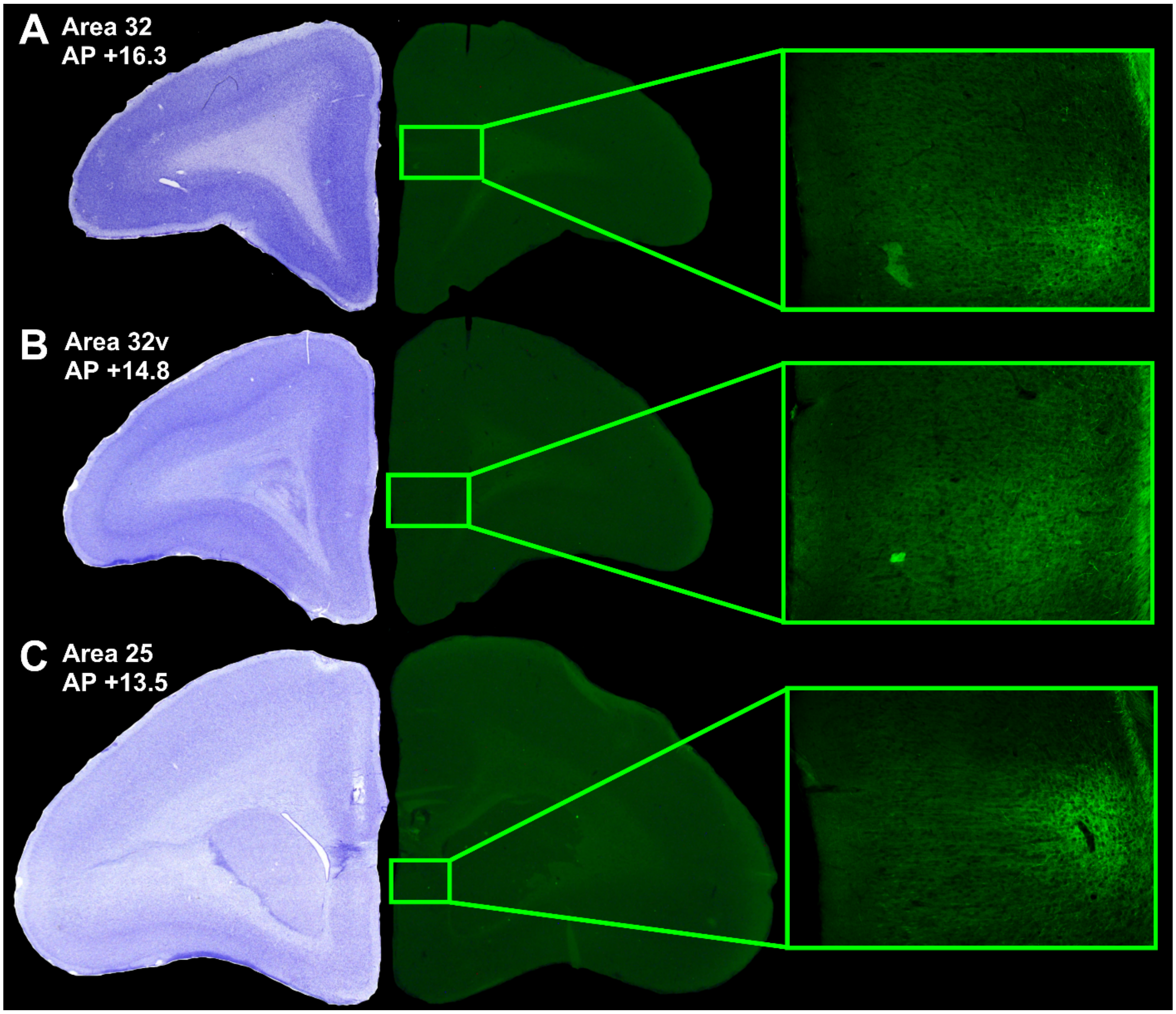

Fig. S6. Projection terminals from A46 within downstream structures A32, A32v and A25. Representative photomicrographs of adjacent cresyl (left) and HA-tag (middle) immunofluorescence sections are placed side by side with high magnification images showing HA-tagged axons and terminals within A32 (**A**), A32v (**B**) and A25 (**C**) on the right. It can be seen that the cresyl sections shown here lie adjacent to those displaying the infusion sites in Fig S5. Anteroposterior co-ordinates for each location are based on the marmoset atlas (*30*).

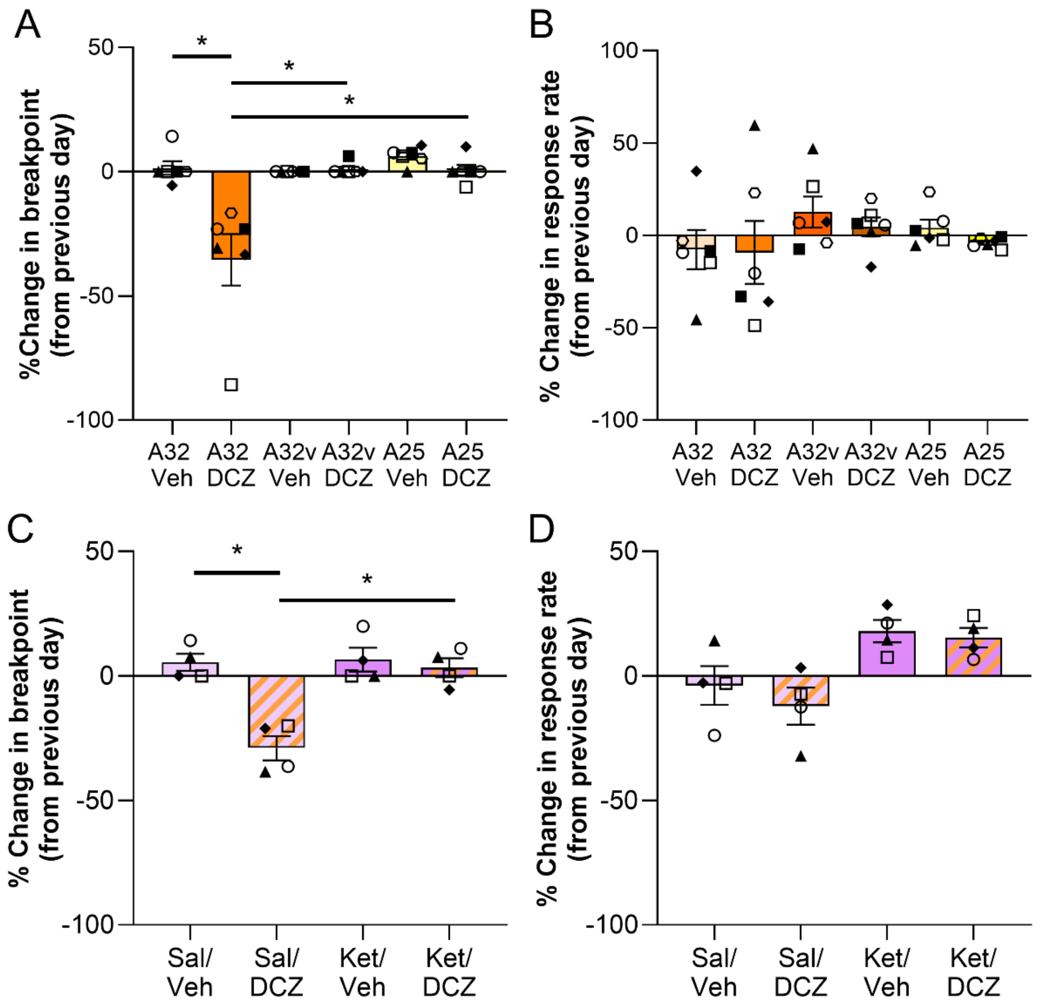

Fig. S7. Infusion of DCZ into A32 reduces breakpoint without impacting response rate, an effect ameliorated by ketamine infusion into A25. (**A**) DCZ infusion into A32 but not A32v or A25 reduced the number of rewards received (breakpoint) on the PR task (n=6, rmANOVA, region*treatment interaction, $F_{(1.14,5.72)}$=13.28, p=0.011, η^2= 0.726; Sidak corrected post-hoc: A32 Veh vs A32 DCZ, p=0.015, *d*=1.48; A32 DCZ vs A32v DCZ, p=0.046, *d*=1.47; A32 DCZ vs A25 DCZ, p=0.037, *d*=1.56). (**B**) This reduction in rewards received occurred without any DCZ infusion significantly impacting the rate of responses during the testing session (rmANOVA, region*treatment interaction F<1, p=0.923). (**C**) Ketamine infusion into A25 blocked the ability of DCZ infusion in A32 from reducing the number of rewards received (n=4, rmANOVA, pretreatment*treatment interaction, $F_{(1,3)}$=18.218, p=0.024; Sidak corrected post-hoc: Saline (Sal)/Vehicle (Veh) vs Sal/DCZ, p=0.024, *d*=2.37; Sal/DCZ vs Ketamine (Ket)/DCZ, p=0.032, *d*=2.09). (**D**) Neither pretreatment with ketamine in A25 or DCZ infusion in A32 impacted the rate of responses during the test session (F<1, p=0.78). Data are displayed as means ± SEM with individual data points with Sidak-corrected post-hoc significance indicated.

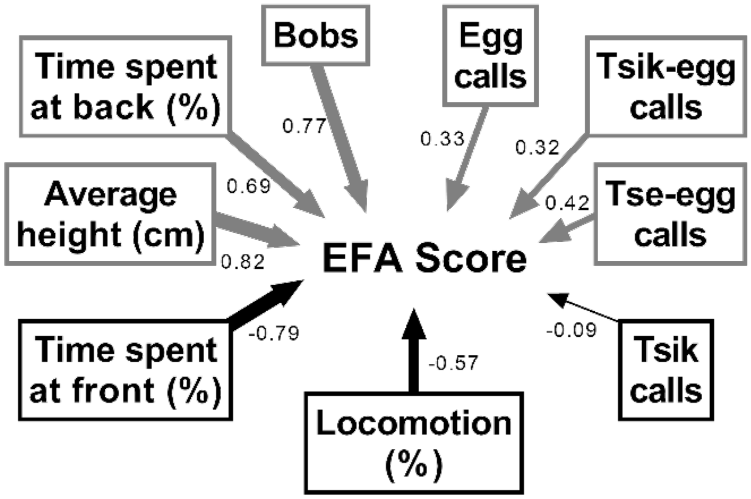

Fig. S8. Exploratory factor analysis loading for threat score in the human intruder test. The factor loading of each measure used in calculating threat reactivity score, which is derived from an exploratory factor analysis from a cohort of 171 marmosets (*31*). Positive weightings are indicated by grey arrows and raise the threat response score, whilst negative weightings are indicated in black and lower the threat response score. Weighting values are indicated for each measure and visualised through thickness of their corresponding arrow.

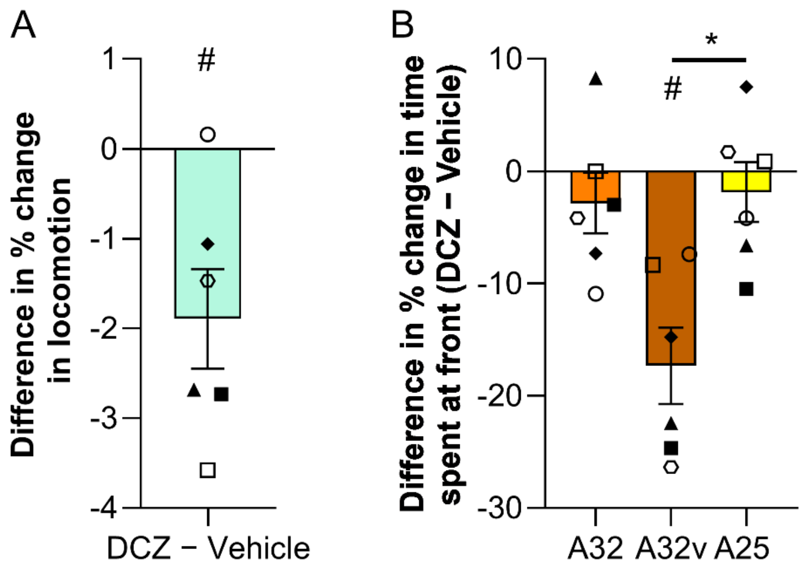

Fig. S9. Individual measures contributing to the heightened threat responses in the human intruder test. (**A**) Systemic DCZ treatment decreased locomotion when compared to vehicle (n=6, one samples t-test, p=0.019, *d*=1.39). (**B**) DCZ infusion into A32v reduced the time spent at the front of the cage compared to vehicle, which was also different to A25 DCZ infusion (n=6, rmANOVA, treatment effect, $F_{(1.7, 8.7)}$=7.70, p=0.014, η^2=0.606; Sidak corrected post-hoc, A32v vs A25, p=0.024, *d*=1.73; one sampled t-test, A32v, p=0.004, *d*=2.08). Data are displayed as means ± SEM with individual data points with Sidak-corrected post-hoc significance indicated by * and significant one sampled t-tests versus a hypothetical mean of 0 indicated by #.

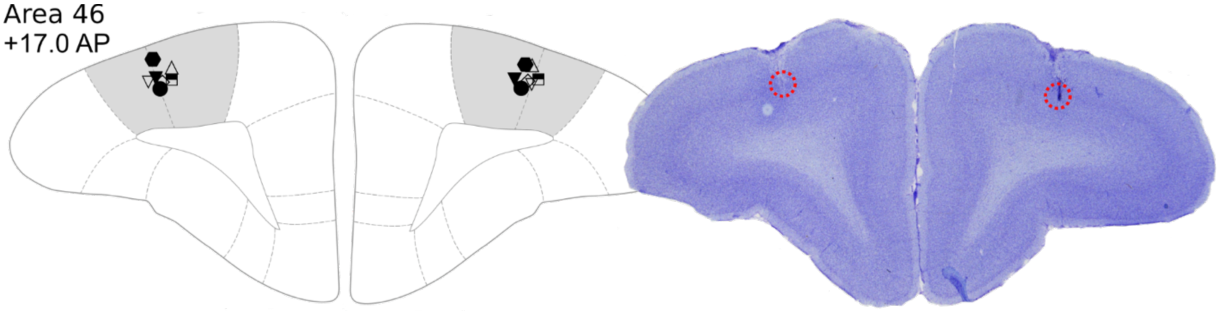

Fig. S10. Histological verification of infusion targeting to Area 46 through intracerebral cannulae. Schematic coronal sections for Area 46 (left) at anteroposterior co-ordinate +17.0mm (*32*) with light grey shading indicating region of interest. Subject-specific symbols indicate individual localisation within each region for each marmoset. Representative cresyl sections are provided for one subject (downward solid triangle) with each location indicated within a dashed red circle.

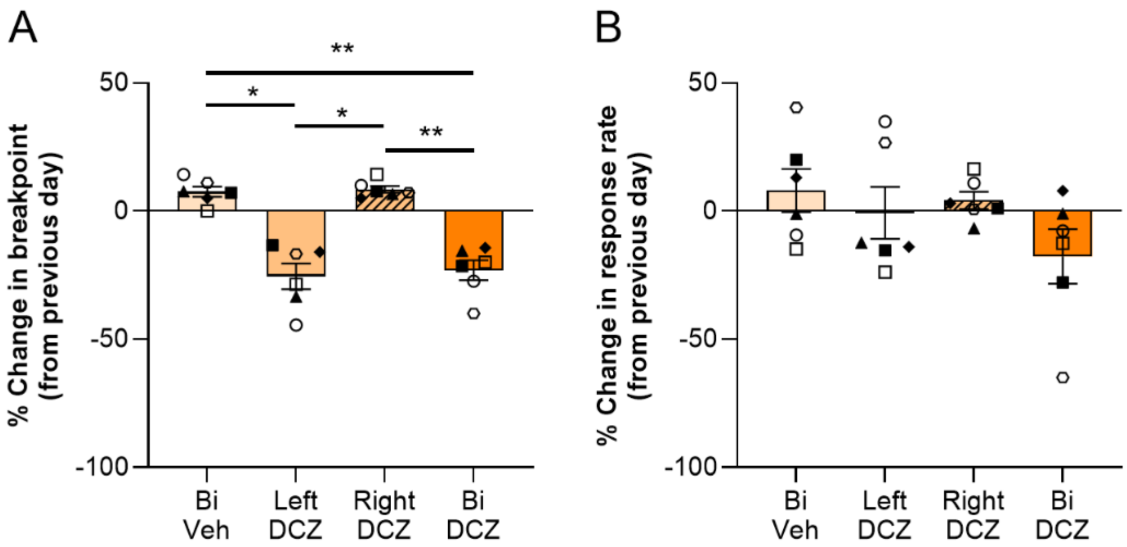

Fig. S11. Functional asymmetry for A46 to A32 projections show left hemispheric involvement in appetitive motivation. (**A**) Infusions of DCZ into left and bilateral (Bi) A32 reduced the number of rewards received (breakpoint), with an infusion into right A32 having no effect ($F_{(3,15)}$=29.925, p<0.001; Sidak-corrected post-hoc comparisons, Bi Veh vs Left DCZ p=0.016, *d*=2.25; Bi Veh vs Bi DCZ p=0.008, *d*=2.36; Left DCZ vs Right DCZ p=0.012, *d*=2.43; Right DCZ vs Bi DCZ p=0.002, *d*=3.06). (**B**) Response rates were unaffected by any unilateral or bilateral DCZ infusion ($F_{(2.89,14.47)}$=1.41, p=0.28). Data are displayed as means ± SEM with individual data points with significant Sidak-corrected post-hoc comparisons indicated.

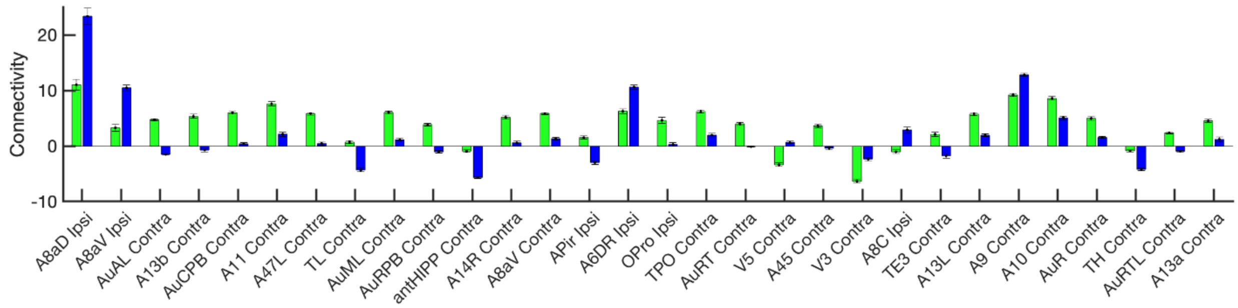

Fig. S12. Mean functional connectivity of the top 30 regions that differentiate the asymmetric hemispheric clusters in marmoset resting state fMRI data. K-means clustering (k=4) of A46 awake resting state fMRI data in 20 marmosets (*33*) show two clusters with biased representation in individual hemispheres, suggestive of asymmetric connectivity (see Fig 4D). Data is displayed for the top 30 regions with largest differences between the asymmetric clusters with right (green) and left (blue) A46. The overall difference between the two clusters lying in the left and right hemispheric of A46, respectively, is the greater connectivity of right A46 with the contralateral side compared to left A46, suggestive of greater overall bilateral connectivity. Bars represent the mean connectivity of all eligible cluster voxels (>75% confidence) with a given region and are displayed as z-statistics and CI as error bars.

Subject/ symbol		Sex	Pre-DREADD analysis	Systemic inactivation	Ketamine amelioration	Pathway analysis	Ketamine pathway amelioration	Asymmetry pharmacology (HI only)	Asymmetry DREADD pathway
1	▲	F		✓	✓	✓	✓		✓
2	♦	M		✓	✓	✓	✓		✓
3	○	F		✓	✓	✓	✓		✓
4	□	M		✓	✓	✓	✓		✓
5	■	F	✓	✓	✓	✓			✓
6	⬡	M	✓	✓	✓	✓			✓
7	◐	F	✓						
8	⧨	M	✓						
9	◇	F						✓	
10	●	M						✓	
11	▼	M						✓	
12	⬢	M						✓	
13	△	F						✓	
14	▽	M						✓	
15	⬒	F						✓	

Table S1. Summary of experimental marmoset involvement. Individual marmoset data symbol, sex and study phase inclusion are indicated across all 15 subjects. 2 subjects were only involved in the pre-virus surgery analysis of the Progressive ratio task. HI = human intruder.

Subject	Early	Middle	Late
1	106.6	114.8	113.4
2	109.4	140.8	125.4
3	54	38.4	38.2
4	142.8	151.4	134.6
5	105	110.2	101.4
6	140.8	160.8	148

Table S2. Average total responses across a week of progressive ratio within early, mid and late phases of the study. Individual marmoset's total responses were averaged across a typical testing week at each of the three phases. No manipulations were undertaken during these weeks. Marmosets display relatively stable performance across the study, as evidenced by a non-significant one-way ANOVA ($F_{(1.23, 6.16)}$=2.46, p=0.17).

Data S1. All data used to create figures within the manuscript (Figs. 1-4) and supplementary figures (figs S1-12). Individual tabs provide the plottable data, with k-means clustering data for Fig. 4D and Fig 4E provided in separate tabs.
